# Yokukansan, a Traditional Japanese Medicine, Enhances the Glutamate Transporter GLT-1 Function in Cultured Rat Cortical Astrocytes

**DOI:** 10.1155/2018/6804017

**Published:** 2018-05-06

**Authors:** Toshiyuki Ueki, Zenji Kawakami, Hitomi Kanno, Yuji Omiya, Kazushige Mizoguchi, Masahiro Yamamoto

**Affiliations:** Tsumura Kampo Research Laboratories, Kampo Research & Development Division, Tsumura & Co., 3586 Yoshiwara, Ami-machi, Inashiki-gun, Ibaraki 300-1192, Japan

## Abstract

Astrocytes carry two glutamate transporters—GLAST and GLT-1—the latter of which is responsible for >90% of glutamate uptake activity in the brain; however, under culture conditions, the GLT-1 expression in astrocytes is exceedingly low, as is the glutamate uptake activity mediated by GLT-1. This study aimed to elucidate the effects of yokukansan (YKS) in relation to the GLT-1-mediated regulation of extracellular glutamate concentrations. Thus, we treated cultured astrocytes with tumor necrosis factor-*α* (TNF-*α*) and dibutyryl-cAMP (dBcAMP) (hereinafter, referred to as “TA”) to increase GLT-1 expression and then functionally examined how YKS would affect glutamate uptake ability derived from GLT-1. Contrary to expectations, although the TA treatments did not affect the uptake activity, YKS significantly augmented it. Conversely, GLAST-derived glutamate uptake was significantly reduced by TA treatments but was unaffected by YKS. Subsequently, we analyzed the GLT-1 protein and mRNA levels and found that TA treatments had significantly increased them, which were then further augmented by YKS. These findings suggest that YKS enhances GLT-1-derived glutamate transport functions in TA-treated cultured astrocytes and that this process entails increased GLT-1 protein and mRNA levels. This type of mechanism may contribute to the YKS-mediated regulation of extracellular glutamate concentrations.

## 1. Introduction

Yokukansan (YKS), a traditional Japanese (Kampo) medicine, is composed of seven dried medicinal herbs. YKS has been approved by the Japanese Ministry of Health, Labour and Welfare as a treatment for neurosis and insomnia and night crying and irritability in children. Recent clinical studies have reported that YKS improves the behavioral and psychological symptoms of dementia (BPSD), such as hallucinations, agitation, aggressiveness, and anxiety, in patients with AD, dementia with Lewy bodies, and other forms of senile dementia [1–4].

Cognitive dysfunction and BPSD are thought to be associated with neurofunctional and neuropathological abnormalities in the brain. In several animal models used to study the pathogenesis of and therapy for dementia, an increase in the extracellular levels of excitatory amino acids, such as glutamate, in the brain has been demonstrated [5–8]. Glutamate is well known to contribute not only to induction of excitation of postsynaptic neurons but also to excitotoxic neuronal death due to the intensity and duration of glutamate exposure [9, 10]. Under physiological conditions, neurotransmission to postsynaptic glutamate receptors is terminated by the clearance of glutamate from the synaptic cleft by transporter proteins located in neuronal and astroglial cells, which prevents glutamate overload in the neurons. In particular, astrocytes play an important role in the efficient removal of glutamate from the extracellular space through glutamate transporters [11, 12]. These findings suggest that failure of the glutamatergic system is related to the appearance of cognitive dysfunction and BPSD. Using an animal model of neurosis and BPSD, we previously demonstrated that YKS ameliorates the degeneration of neuronal and astroglial cells in the brain stem, cerebral cortex, and hippocampus of rats subjected to thiamine deficiency (TD) [13, 14]. These ameliorations were responsible for reducing agitation, aggressiveness, and various psychological functions [13]. Moreover, it was confirmed that an increase in the extracellular level of glutamate in the brain and YKS inhibits this increase [13].

Five different isoforms of glutamate transporters (or excitatory amino acid carriers) have been identified: glutamate-aspartate transporter (GLAST, EAAT1), glutamate transporter 1 (GLT-1, EAAT2), EAAC-1 (EAAT3), EAAT4, and EAAT5 [15–19]. GLT-1 and GLAST are enriched in the astrocytes, EAAC1 and EAAT4 are enriched in neurons, and EAAT5 is enriched in the retina [20, 21]. In astrocytes, GLAST is the predominant glutamate transporter during brain development, whereas GLT-1 becomes the most abundant transporter in mature organisms and is responsible for up to 90% of the total glutamate clearance in adult tissues [22]. The results of our previous study showed that YKS ameliorated not only the TD-induced reduction of glutamate uptake through GLAST but also the decreases in GLAST proteins in cultured astrocytes [23]; however, the effects of YKS on the function and protein levels of GLT-1 remain unknown. Recently, in mice, YKS protected against the decrease in hippocampal GLT-1 expression induced by chronic restraint stress [24]. In contrast, no changes were observed in the levels of GLAST, EAAC-3, or EAAT4 in stress-maladaptive mice [24]; however, whether this protective effect on GLT-1 was caused by direct or indirect actions of YKS is unclear.

In pure astrocyte cultures, glutamate uptake is largely dependent on GLAST activity which prevails over GLT-1 [23, 25], suggesting that normal cultured astrocytes are inappropriate to evaluate the drugs on glutamate uptake ability through GLT-1. Several* in vitro* studies suggest that inflammatory mediators regulate glutamate transporters in cultured astrocytes [26]. In several models, dibutyryl-cAMP- (dBcAMP-) exposed astrocytes showed enhanced expression of both GLAST and GLT-1 [25, 27]. In addition, in dBcAMP-exposed astrocytes, tumor necrosis factor-alpha (TNF-*α*), a key inflammatory cytokine, induced a robust upregulation of GLT-1 proteins [28].

The present study aimed to clarify the effect of YKS on GLT-1 function and its protein and mRNA expression levels. As described above, compared with that of GLAST, GLT-1 expression is very low in cultured astrocytes; therefore, we focused on GLT-1 by treating cultured astrocytes with TA. First, we attempted to enhance the GLT-1-derived glutamate uptake ability in cultured astrocytes by treating with TA. Second, we examined the augmentative effect of YKS on glutamate uptake by GLT-1 in cultured astrocytes treated with TA. Last, we explored the mechanism underlying the effect of YKS by investigating the changes in expression of both the mRNA and protein levels in cultured astrocytes treated with TA.

## 2. Materials and Methods

### 2.1. Drugs and Reagents

YKS used in the present study was a dry powdered extract from a mixture of* Atractylodes lancea *rhizome (4.0 g, rhizome of* Atractylodes lancea* De Candolle), Hoelen (4.0 g, sclerotium of* Poria cocos* Wolf), Cnidii Rhizoma (3.0 g, rhizome of* Cnidium officinale* Makino), Uncaria thorn (3.0 g, thorn of* Uncaria rhynchophylla* Miquel), Japanese Angelica root (3.0 g, root of* Angelica acutiloba* Kitagawa), Bupleurum root (2.0 g, root of* Bupleurum falcatum* Linné), and Glycyrrhiza root (1.5 g, root and stolon of* Glycyrrhiza uralensis* Fisher) that was supplied by Tsumura & Co. (Tokyo, Japan).

Reagents used in the cell culture experiments, dehydrokainate (DHK), pyrithiamine hydrobromide, Dulbecco's modified Eagle's medium (DMEM), DNase, glutamate dehydrogenase, *β*-nicotinamide adenine dinucleotide, 1-methoxyphenazine methosulphate, Triton X-100, TNF-*α*, and dBcAMP were purchased from Sigma-Aldrich (St. Louis, MO, USA). EDTA, HEPES, and MTT were purchased from Dojindo (Kumamoto, Japan). Purified rabbit polyclonal antiserum to glial fibrillary acidic protein (GFAP) was purchased from Chemicon (Temecula, CA, USA). VECTASTAIN Elite ABC (rabbit immunoglobulin [Ig]-G) and DAB substrate kits for peroxidases were purchased from Vector Laboratories (Burlingame, CA, USA). Other chemicals were purchased from commercial sources.

For Western blotting, a protease inhibitor cocktail, blocking buffer (SuperBlock), and Can Get Signal Solutions were purchased from Sigma-Aldrich (St. Louis, MO, USA), Thermo Fisher Scientific (Rockford, IL, USA), and Toyobo (Osaka, Japan), respectively. Rabbit anti-GLT-1 was purchased from Frontier Institute (Hokkaido, Japan). Rabbit anti-GAPDH was purchased from Cell Signaling Technology Japan (Tokyo, Japan). Sheep anti-rabbit horseradish peroxidase- (HRP-) conjugated secondary antibodies were purchased from GE Healthcare (Buckinghamshire, England). Pierce Western Blotting Substrate Plus was obtained from Thermo Fisher Scientific. The protein assay kit using the Lowry method was purchased from Bio-Rad Laboratories (Hercules, CA, USA).

For the real-time reverse-transcription polymerase chain reaction (real-time RT-PCR) assay, a Qiagen RNeasy mini kit was purchased from Qiagen (Hilden, Germany), and a High Capacity cDNA Reverse Transcription Kit, TaqMan Gene Expression Master Mix, and TaqMan gene expression assay probes for the detection of GLT-1, GLAST, and Rps29 were purchased from Applied Biosystems (Thermo Fisher Scientific).

### 2.2. Preparation of Primary Astrocyte Culture

Astrocytes were cultured using a previously described procedure [23, 29]. In brief, forebrain cortices were dissected from 0-1-day-old Sprague Dawley rats and the meninges carefully removed. Neopallia were mechanically disrupted by pipetting into them DMEM/Ca- and Mg-free phosphate buffered saline [PBS(−)] (1 : 1). After filtering with 100 *μ*m mesh (BD Falcon, Bedford, MA, USA) and lens-cleaning paper (Fujifilm, Tokyo, Japan), the cells (5.0 × 10^6^) were seeded into a 75 cm^2^ culture flask (Corning, Corning, NY, USA) with DMEM containing 7.5 mM glucose, 2 mM glutamine, 25 mM NaNCO_3_, and 10% horse serum. The next day, the culture medium was exchanged with DMEM containing 25 mM sorbitol, 2 mM glutamine, 25 mM NaNCO_3_, and 10% dialyzed horse serum, and the cells were maintained in a humidified, 5% CO_2_ incubator at 37°C for 2 weeks. After the incubation period, the cultures were returned to the glucose-containing DMEM medium. Approximately 95% of the cells were positively immunoreactive to an astrocyte marker, GFAP.

The high purity-cultured astrocytes were seeded at approximately 20,000 cells/cm^2^ into Primaria culture plates (Becton Dickinson, Franklin Lakes, NJ, USA) and used in the following experiments after they reached confluency at ~5 d.

### 2.3. Examination of the Effects of YKS on Glutamate Uptake in Cultured Astrocytes

The astrocytes in the 96-well plate were treated with 50 ng/mL TNF-*α* and/or 150 *μ*M dBcAMP to induce GLT-1. The cultures were then treated with YKS (final concentration: 500 *μ*g/mL), which was filtered through a 0.22 *μ*m filter.

Glutamate uptake ability was evaluated in cells cultured for 3 d. We followed the methods of Kawakami et al. [23]. Namely, 100 *μ*M glutamate was exogenously added to the astrocyte cultures. The aliquot of the culture medium was carefully collected after incubation for 3 h. DHK, a specific inhibitor of GLT-1, was added with the glutamic acid. The extracellular concentration of glutamate was determined using the modified colorimetric method previously described [30]. In brief, 50 *μ*L of the medium was mixed with the same volume of substrate mixture comprising 20 U/mL glutamate dehydrogenase, 2.5 mg/mL nicotinamide adenine dinucleotide, 0.25 mg/mL MTT, 100 *μ*M 1-methoxyphenazine methosulfate, and 0.1% Triton X-100 in 0.05 M Tris-HCl buffer; pH 8.2. The mixture was incubated at 37°C, and the reaction was stopped after 10 min by adding 100 *μ*L of a stop solution, pH 4.7, containing 50% dimethylformamide and 20% sodium dodecyl sulfate (SDS). The amount of MTT formazan produced was determined by measuring absorbance using a microplate reader at a test wavelength of 540 nm and a reference wavelength of 690 nm. The glutamate concentrations in the samples were estimated from the standard curve that was constructed for each assay using cell-free medium containing known concentrations of L-glutamate.

### 2.4. Examination of the Effects of YKS on Glutamate Transporter Proteins in Cultured Astrocytes

The astrocytes were prepared according to the procedure described above. In this experiment, GLT-1 proteins in the astrocytes that were cultured for 3 d were analyzed using Western blotting. In brief, the astrocytes were rinsed and scraped in PBS (−). After centrifugation at 200 ×g for 15 min at 4°C, cells were solubilized in lysis buffer containing 50 mM Tris, 150 mM NaCl, 0.1% SDS, 1% Nonidet P-40, 0.5% sodium deoxycholate (pH 8.0), and a protease inhibitor cocktail. The cell lysate was then incubated for 30 min at 4°C on a rotating device (PVM-2000; LMS, Tokyo, Japan), followed by centrifugation at 22,000 ×g for 15 min to yield a whole cellular fraction containing cytoplasm and plasma membranes, but not nuclei. After measuring the protein concentration, the samples (0.3 *μ*g/lane) were subjected to SDS-10% polyacrylamide gel electrophoresis and transferred to polyvinylidene difluoride membranes (Hybond-P, GE Healthcare). Nonspecific binding was blocked by incubating with SuperBlock blocking buffer at room temperature for 1 h, followed by incubating overnight at 4°C in Can Get Signal Solution 1 (Toyobo) with rabbit anti-GLT-1 (1 : 6,000), or rabbit anti-GAPDH (1 : 10,000). The blots were washed and incubated for 1 h at room temperature in Can Get Signal Solution 2 (Toyobo) with sheep anti-rabbit HRP-conjugated secondary antibody (1 : 25,000). The HRP activity was visualized by Pierce Western Blotting Substrate Plus using the Typhoon Imaging System (GE Healthcare).

### 2.5. Examination of the Effects of YKS on GLT-1 mRNAs in Cultured Astrocytes

The astrocytes were prepared according to the procedure described above. They were cultured for 3 d, and either GLT-1 mRNA or GAPDH mRNA as the reference gene was determined by real-time RT-PCR. In brief, total RNA from the astrocytes was extracted using a Qiagen RNeasy micro kit (Qiagen, Hilden, Germany) according to the manufacturer's instructions. The concentration of extracted total RNA was determined spectrophotometrically at 260 nm. Reverse transcription was performed using a High Capacity cDNA Reverse Transcription Kit (Thermo Fisher Scientific) according to the manufacturer's instructions on a TAK-TP400 Thermal Cycler (Takara Bio Inc., Shiga, Japan). Next, real-time PCR was performed using TaqMan Gene Expression Master Mix (Thermo Fisher Scientific). PCR was run on an ABI Prism 7900HT sequence detection system with a 384-well format (Thermo Fisher Scientific). TaqMan gene expression assay probes used were GLT-1 (Rn00568080_m1) and GAPDH (Rn01775763_g1). Real-time PCR was performed in triplicate for each sample. Differences in amplification were determined using the 2^−ΔΔCT^ method. GAPDH was used as an endogenous control to normalize expression levels between samples.

### 2.6. Statistical Analysis

Data are presented as the mean ± SEM. The statistical significance of differences between groups in the cell culture experiments was assessed by one-way analysis of variance followed by the Bonferroni multiple comparisons test. The significance level in each statistical analysis was *P* < 0.05.

## 3. Results

### 3.1. Effect of the TA Treatments and YKS on Glutamate Uptake in Astrocytes

**Figure 1:**
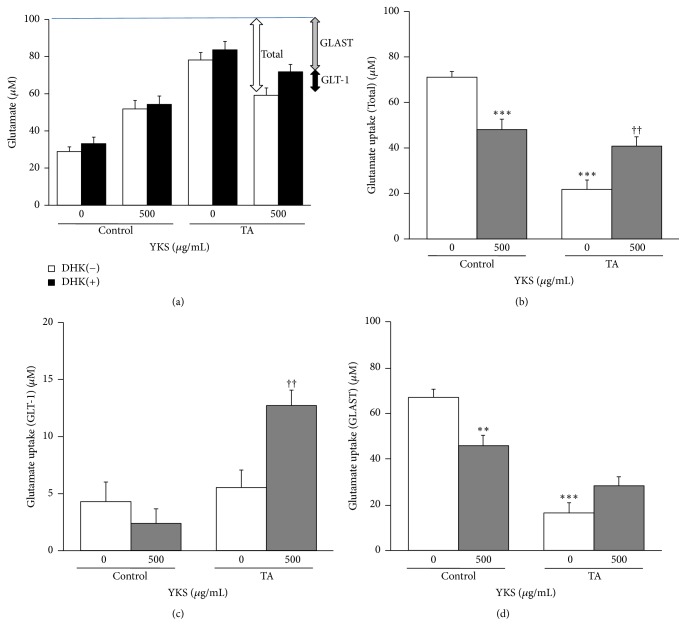
*Effect of TA treatments and Yokukansan (YKS) on glutamate uptake in cultured rat cortical astrocytes*. Astrocytes were exposed for 72 h in culture medium supplemented with or without 50 ng/mL TNF-*α* and 150 *μ*M dBcAMP (TA). The cultures were treated with or without 500 *μ*g/mL YKS. One day before measuring glutamate uptake, the content was replaced with serum-free medium. The glutamate uptake ability in each sample was evaluated based on the glutamate concentration measured in the culture supernatant collected from the culture medium 3 h after adding 100 *μ*M glutamate (a). Here, the total glutamate uptake (total) is represented as the difference between the 100 *μ*M glutamate added and the glutamate concentration in the culture medium. The difference between the glutamate concentration in the medium containing DHK (DHK (+)) and the glutamate concentration in the DHK-free medium (DHK (−)) signifies the GLT-1-mediated glutamate uptake, whereas the difference between the 100 *μ*M added glutamate and DHK (+) is regarded as the GLAST-mediated glutamate uptake. (b), (c), and (d) show the total, GLT-1-mediated, and GLAST-mediated glutamate uptakes, respectively. Each column is the mean ± SEM (*n* = 15). Asterisks and daggers indicate a significant difference: ^*∗∗*^*P* < 0.01, ^*∗∗∗*^*P* < 0.001 versus Control (0 *μ*g/mL YKS), and ^††^*P* < 0.01 versus TA (0 *μ*g/mL YKS).

We examined the effects of TA treatments and YKS on glutamate uptake (Figure 1). The total glutamate uptake is represented as the difference between the added glutamate concentration (100 *μ*M) and the glutamate concentration in the culture medium. The GLT-1-mediated glutamate uptake is expressed as the difference between the glutamate concentration in the culture medium to which DHK (a GLT-1 inhibitor) was added (DHK-added medium) and the glutamate concentration in the DHK-free medium (control) (Figures 1(a) and 1(c)). The GLAST-mediated glutamate uptake is calculated as the difference between the added glutamate concentration and the glutamate concentration in the medium containing DHK (Figures 1(a) and 1(d)). Total glutamate uptake (Figure 1(b)) was significantly reduced by TA treatments compared with untreated TA (*F*(3, 56) = 28.050, *P* < 0.001), whereas YKS significantly compensated for that reduction (*F*(3, 56) = 28.050, *P* < 0.01). However, for untreated TA, YKS was significantly decreased conversely (*F*(3,56) = 28.050, *P* < 0.01). Of the components of this total glutamate uptake, GLT-1-mediated uptake was not affected by TA treatments compared with untreated TA (Figure 1(c)) but was significantly augmented by YKS (*F*(3,56) = 9.274, *P* < 0.01). In contrast, GLAST-mediated uptake (Figure 1(d)) was significantly reduced by TA treatments compared with untreated TA (*F*(3,56) = 28.041, *P* < 0.001), and YKS did not offset this reduction. However, for untreated TA, YKS was significantly decreased conversely (*F*(3,56) = 28.041, *P* < 0.01).

### 3.2. Effect of TA Treatments and YKS on Glutamate Transporter GLT-1 Protein Expression in Astrocytes

**Figure 2:**
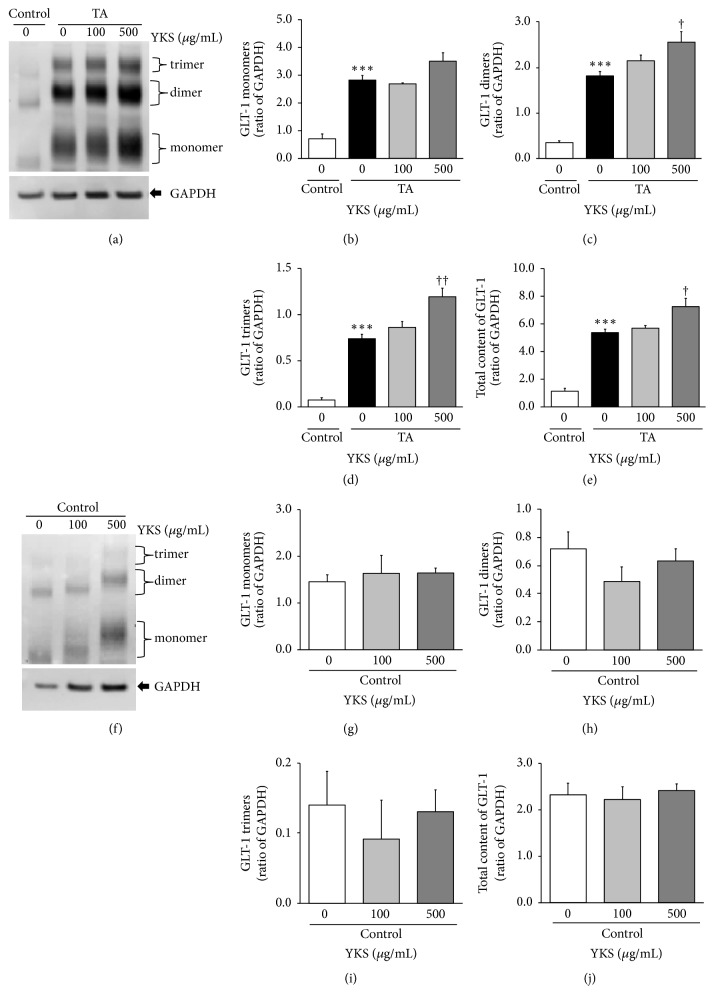
*Effect of TA treatments and Yokukansan (YKS) on glutamate transporter GLT-1 protein expression in cultured rat cortical astrocytes*. Astrocytes were exposed for 72 h in culture medium supplemented with or without 50 ng/mL TNF-*α* and 150 *μ*M dibutyryl-cAMP (TA) and then treated with or without 100 or 500 *μ*g/mL YKS. The relative expression of GLT-1 protein was examined using Western blotting. Cell lysates contained cytoplasm and plasma membrane but not nuclei. (a, f) Representative images of Western blots. Immunoreactive GLT-1 was detected as monomer (70 kD), dimer (140 kD), and trimer (210 kD) bands. Immunoreactive GAPDH was detected as single bands (37 kD). (b–e, g–j) Results of densitometric analysis in monomers (b, g), dimers (c, h), trimers (d, i), and total (e, j). Each column is the mean ± SEM (*n* = 3-4). Asterisks and daggers indicate a significant difference: ^*∗∗∗*^*P* < 0.001 versus control (0 *μ*g/mL YKS), ^†^*P* < 0.05, and ^††^*P* < 0.01 versus TA (0 *μ*g/mL YKS).

We then examined the effects of TA treatments and 100 and 500 *μ*g/mL YKS on GLT-1 protein expression within astrocyte lysate (Figure 2). GLT-1 was detected as oligomers: a monomer at ~70 kD, a dimer at ~140 kD, and a trimer at ~210 kD on the electrophoresis gel (Figures 2(a) and 2(f)). Although these oligomers were expressed in extremely small quantities, TA treatments significantly increased the expression levels (monomer (Figure 2(b): *F*(3,11) = 34.525, *P* < 0.001), dimer (Figure 2(c): *F*(3,11) = 45.847, *P* < 0.001), trimer (Figure 2(d): *F*(3,11) = 58.566, *P* < 0.0001), and total (Figure 2(e): *F*(3,11) = 52.970, *P* < 0.001). Compared to TA treatments alone, 500 *μ*g/mL YKS significantly augmented the expression levels of the GLT-1 dimer (Figure 2(c): (3,11) = 45.847, *P* < 0.05), trimer (Figure 2(d): *F*(3,11) = 58.566, *P* < 0.01), and total content (Figure 2(e): *F*(3,11) = 52.970, *P* < 0.05). On the other hand, YKS did not affect the expression levels of any oligomers against untreated TA (Figures 2(f)–2(j)).

### 3.3. Effect of TA Treatments and YKS on Glutamate Transporter GLT-1 mRNA Expression in Astrocytes

**Figure 3:**
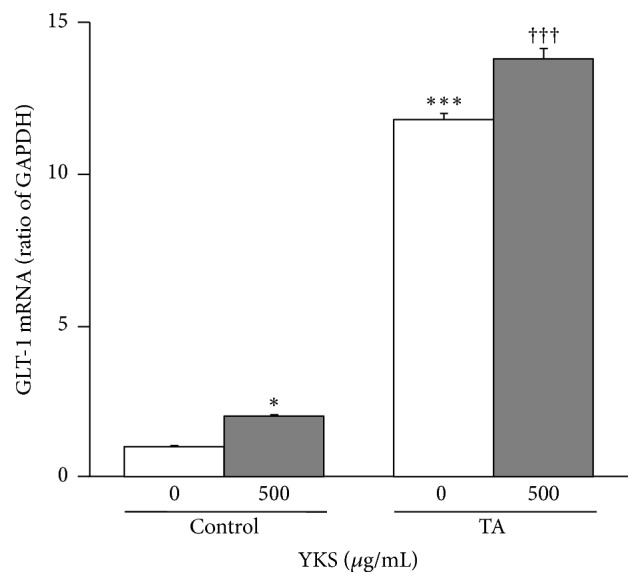
*Effect of TA treatments and Yokukansan (YKS) on glutamate transporter GLT-1 mRNA expression in cultured rat cortical astrocytes*. Astrocytes were exposed for 72 h with culture medium supplemented with or without 50 ng/mL TNF-*α* and 150 *μ*M dibutyryl-cAMP (TA). At the same time, the cultures were treated with or without (control) 500 *μ*g/mL YKS. Each column is expressed as a relative value of the control and mean ± SEM (*n* = 3). Asterisks indicate a significant difference: ^*∗*^*P* < 0.05, ^*∗∗∗*^*P* < 0.001 versus control (0 *μ*g/mL YKS), and ^†††^*P* < 0.001 versus TA (0 *μ*g/mL YKS).

We examined the effects of TA treatments and 500 *μ*g/mL YKS on the expression of GLT-1 mRNA in astrocytes (Figure 3). TA treatments significantly increased the expression of GLT-1 compared to that in the control (*F*(3,8) = 1085.214, *P* < 0.001). Compared to TA treatments alone, 500 *μ*g/mL YKS significantly augmented GLT-1 expression (*F*(3,8) = 1085.214, *P* < 0.001). YKS also significantly enhanced the expression level of GLT-1 against untreated TA (*F*(3,8) = 1085.214, *P* < 0.05).

## 4. Discussion

The present study reveals the following two new findings: (1) YKS augments GLT-1-derived glutamate uptake in TA-treated, but not untreated astrocytes, and (2) YKS further boosts the expression of GLT-1 protein and mRNA that were increased by TA treatments. The present study aimed to functionally analyze whether YKS would complement GLT-1-derived glutamate uptake, thereby elucidating the mechanism of action for that process. In previous studies [13, 14, 23], we reported that YKS rectifies the following thiamine deficiency-induced abnormalities in the brain and cultured astrocytes of rats: astrocyte degeneration, increase in extracellular glutamate concentrations, decrease in GLAST-derived glutamate uptake, and reduced expression of GLAST proteins and mRNA. In the present study, we focused on GLT-1, another glutamate transporter that is predominantly expressed in astrocytes. First, we had to establish a line of cultured astrocytes that would enable us to evaluate GLT-1-derived glutamate uptake. In the cultured astrocytes, GLT-1-derived glutamate uptake increases through either coculture with nerve cells or the addition of a culture solution containing LPS-activated microglia. It also increases when astrocytes are cultured in the presence of dBcAMP, a cell-permeable cAMP analog, and TNF-*α* or in the presence of both TNF-*α* and cAMP [12, 25, 28]. In a preliminary experiment, we treated cultured astrocytes with only TNF-*α*, only dBcAMP, or TA (a combination of both factors) for 3 d and observed changes in the expression of GLT-1 mRNA. Consequently, we found that the highest levels of expression were achieved when the cells were subjected to TA treatments (data not shown).

We then examined the changes in glutamate uptake levels in TA-treated astrocytes. Contrary to our expectations, the TA-treated astrocytes did not exhibit significant changes in GLT-1-derived glutamate uptake compared to that in the control. Under these conditions, GLT-1-derived glutamate uptake increased in the TA-treated astrocytes only when YKS was added (Figure 1(c)).

Subsequently, Western blotting of the soluble fraction of the astrocytes (i.e., that containing plasma membrane and cytoplasmic proteins) detected GLT-1 as a monomer or oligomer (dimer or trimer) (Figures 2(a) and 2(f)). It was revealed that TA stimulation significantly increased the expression levels of GLT-1 proteins in all these supramolecular forms. This result is partially consistent with a result obtained in a previous study [28]; however, it is incongruous with the data that indicate that GLT-1-derived glutamate uptake remained unchanged (Figure 1). Tilleux et al. [28] measured uptake levels of [^3^H]aspartate, a substance analogous to glutamate. Once absorbed into astrocytes, [^3^H]aspartate is not excreted extracellularly. In the present study, the glutamate uptake was calculated as the difference between the concentration of glutamate added and the concentration of glutamate present in the culture medium. As such, if glutamate that is absorbed into the astrocytes is discharged into the medium because of factors pertaining to TA treatments, the calculated value will not correctly reflect the actual glutamate uptake volume, making it impossible to accurately gauge the increase, if any, in GLT-1 proteins. Alternatively, it is also possible that TA treatments itself increase GLT-1 proteins but that the larger numbers of GLT-1 proteins are unable to migrate into the plasma membrane, or if they are able, they remain inactive in the membrane.

In the drug treatment study, the expression levels of all GLT-1 supramolecular forms from TA treatments were augmented by adding 500 *μ*g/mL YKS, with the increases in the dimer and trimer expressions being statistically significant. A similar trend was observed with the total expression of GLT-1. Because GLT-1 is expressed as a homotrimer in the plasma membrane, its active conformation is considered to be the trimer [31]. We believe it to be reasonable that YKS augmented glutamate uptake under TA stimulation and further boosted the expression of GLT-1 trimer (Figures 1 and 2).

To determine whether the increased expression of GLT-1 proteins from TA stimulations and YKS was effectuated at the transcription level, we performed an RT-PCR analysis of GLT-1 mRNA. Consequently, we discovered that the expression of GLT-1 mRNA was significantly higher in the TA-treated cells than in the control samples. This result is also consistent with a previous report [28]. These findings signify that both TA stimulation and YKS treatment increase the expressions of GLT-1 proteins and mRNA in an almost parallel manner and hence suggests that both TA stimulation and YKS treatment augment the expression of GLT-1 proteins by promoting the transcription of GLT-1 mRNA.

There is a discrepancy among our data. Thus, TA treatments upregulated GLT-1 proteins but did not increase the GLT-1-derived glutamate uptake activity, as described above. On the other hand, TA treatments concomitant with YKS further upregulated GLT-1 proteins and significantly increased the GLT-1-derived glutamate uptake activity. Although the reasons for this discrepancy remain unknown, YKS may regulate GLT-1 functions through some mechanisms such as glutamate transporting mechanisms, trafficking GLT-1 proteins from the cytoplasm to the plasma membrane, and the internalization of the proteins from the plasma membrane, which should be clarified in future.

In animals, Miyagishi et al. [24] reported that YKS significantly increased GLT-1 proteins specific to the hippocampus in mice with stress adjustment disorder who exhibited elevated levels of emotional behavior. This finding suggests that YKS has the potency to increase GLT-1 proteins* in vivo*. Given this report and the results of the present study, it is possible that certain active ingredients contained in YKS might directly act on hippocampal astrocytes; however, little is known about how YKS functions to augment the expression of GLT-1 mRNA.

Previous pharmacological studies using genetic inhibitors implied that TNF-*α* stimuli activate NF*κ*B through TNF receptor 1-associated death domain protein (TRADD) and dBcAMP stimuli activate NF*κ*B through PI-3K, respectively [32–36]. It was later revealed that NF*κ*B activates the transcription of the GLT-1 promoter by directly binding to it [34, 37]. In the present study, TA treatments plus YKS augmented the expressions of GLT-1 mRNA and proteins. This result suggests the possibility that certain ingredients contained in YKS act on the relevant signal transduction pathways to activate transcription.

In a mice model of temporal lobe epilepsy (TLE), Hsp90*β* increases in reactive astrocytes in the hippocampus, and a bond between Hsp90*β* and GLT-1 accelerates the degradation of GLT-1 mediated by the 20S proteasome. Furthermore, systemic administration of 17-allylamino-17-demethoxygeldanamycin (17AAG), an Hsp90 inhibitor, was reported to increase GLT-1 proteins by inhibiting its proteolysis, thereby improving symptoms of TLE [38]. Moreover, a study using GFAP-null animals revealed that a loss of GFAP leads to a decline in GLT-1-derived glutamate uptake, GLT-1 protein expression, and transportation of GLT-1 into the plasma membrane [39]. It is possible that YKS acts on these factors to augment GLT-1 protein expression and accelerate GLT-1-derived glutamate uptake. As described above, several factors come into play in the expression, intramembrane transportation, and preservation of GLT-1 mRNA and proteins. Further research is needed to elucidate the exact mechanism by which YKS augments GLT-1-derived glutamate uptake activity.

Researchers have been unable to identify galenical preparations or the active ingredients in YKS that are responsible for increasing GLT-1 mRNA expression; however, there are some possible candidates based on the available data. For example, glycyrrhizin, contained in Glycyrrhiza that is one of the constituent herbs, and 18*β*-glycyrrhetinic acid, a metabolite of glycyrrhizin, have been reported to inhibit PKC activity [40]. When astrocytes are stimulated with PMA, a phorbol ester, PKC is activated, resulting in a decline in GLT-1-derived glutamate uptake. In addition, GLT-1 in the plasma membrane decreases from accelerated internalization. For this internalization, the C-terminus of GLT-1 protein must be ubiquitinated [41–43]. In addition to increasing GLT-1 protein expression, YKS suppresses the internalization of GLT-1 found in the plasma membrane by inhibiting the ubiquitination of its C-terminus. This process might enhance GLT-1-derived glutamate uptake activity. Further research is also needed to identify the active ingredients involved in this process.

The findings in the present study suggest that YKS augments the functions of GLT-1, a transporter involved in the protection of nerve cells from an excess of extracellular glutamate, and that a part of this augmentation activity is accompanied by increased expressions of GLT-1 protein and mRNA. As a traditional Japanese medicine, YKS comprises multiple galenical preparations, each of which contains various ingredients. More in-depth research is needed to elucidate the underlying mechanisms for augmenting GLT-1 by YKS.

## 5. Conclusion

It is suggested that YKS augments GLT-1-derived glutamate transport functions in TA-treated cultured astrocytes and that this process involves increased expressions of GLT-1 proteins and mRNA. This study implies that YKS can at least activate GLT-1 in the astrocytes, thereby contributing to the regulation of extracellular glutamate concentrations.
